# Deep learning augmented microscopy: a faster, wider view, higher resolution autofluorescence-harmonic microscopy

**DOI:** 10.1038/s41377-022-00801-z

**Published:** 2022-04-24

**Authors:** Lei Tian

**Affiliations:** 1 Department of Electrical and Computer Engineering, Boston University, Boston, MA 02215 USA; 2 Department of Biomedical Engineering, Boston University, Boston, MA 02215 USA

**Keywords:** Multiphoton microscopy, Imaging and sensing

## Abstract

Deep learning enables bypassing the tradeoffs between imaging speed, field of view, and spatial resolution in autofluorescence-harmonic microscopy.

Label-free nonlinear optical microscopy is an emerging technique for probing biological structures and functions without exogenous labels or dyes. Thus, it provides an attractive solution to gain biological insights without perturbing the native states of biological samples and processes. For example, vibrational spectroscopic imaging techniques^1^, such as anti-Stokes Raman scattering (CARS) and stimulated Raman scattering (SRS), allow quantifying biomolecules by measuring the molecular vibration spectra in living cells and tissues. Two-photon autofluorescence (2PA) of flavin adenine dinucleotide (FAD) and three-photon autofluorescence (3PA) of nicotinamide adenine dinucleotide (NADH) permit noninvasive monitoring of metabolic activities^2^. Second-harmonic generation (SHG) microscopy provides morphological and functional characterization of anisotropic biological structures, such as collagen^3^, muscle^4^, and microtubules^5^. Third harmonic generation (THG) microscopy enables elucidating on cellular and tissue organizations by probing intra- and extracellular membranes, and extracellular matrix structures^6^. Recent advances in simultaneous label-free autofluorescence- multiharmonic (SLAM) microscopy further expands the nonlinear optical microscopy’s utility in intravital imaging^7^ and slide-free, stain-free histopathology^8,9^.

Despite these advances, the implementations of all nonlinear optical microscopy techniques rely on laser scanning. This makes it challenging to simultaneously achieve a wide field of view (FOV), high spatial resolution, and fast imaging speed with sufficient signal-to-noise ratio (SNR) in the measurement. In this work by Shen et al.^10^, a novel deep learning augmented microscopy framework is developed to overcome the physical tradeoffs. The proposed “deep learning autofluorescence-harmonic microscopy” (DLAM) is demonstrated on human pathological tissues for multimodal imaging, including 2PA of FAD, SHG and 3PA of NADH, with enhanced spatial resolution and much reduced acquisition time. This advancement may find broad utilities in the studies of biology and neuroscience.

Broadly speaking, DLAM adds to the rapid-growing list of deep learning augmented microscopy techniques^11^, which overcome different aspects of physical limitations by combining novel instrumentation and deep learning. For example, strategies have been developed to first acquire low-SNR images at high speed and low light exposure and later enhance the SNR by deep learning to alleviate photo-damages in fluorescence microscopy^12^ and SRS microscopy^13^. Deep learning-based super-resolution reconstruction has been demonstrated to bypass the limitation of FOV^14^. Data-efficient acquisition schemes by deep learning have been developed for multi-shot quantitative imaging techniques, such as Fourier ptychographic microscopy^15^, single molecule localization microscopy^16^, and structured illumination microscopy^17^. We envision this deep learning augmented approach may fundamentally push the imaging limits and ultimately revolutionize the field of microscopy.
